# Novel Variation in Acyl-CoA Synthetase Long Chain Family Member 6 (ACSL6) Results in Protein Structural Modification and Multiple Non-Related Neoplasia in a 46-Year-Old: Case Report

**DOI:** 10.3389/fonc.2022.899579

**Published:** 2022-06-02

**Authors:** María Isabel Castillo, Erick Freire, Vanessa I. Romero, Benjamín Arias-Almeida, Carlos Reyes, Kazuyoshi Hosomichi

**Affiliations:** ^1^ School of Medicine, Universidad San Francisco de Quito, Quito, Ecuador; ^2^ Centro de Investigación Biomédica, Facultad de Ciencias de la Salud Eugenio Espejo, Universidad UTE, Quito, Ecuador; ^3^ Departamento de Genetica, Hospital de Especialidades Eugenio Espejo, Quito, Ecuador; ^4^ Department of Bioinformatics and Genomics, Kanazawa University, Kanazawa, Japan

**Keywords:** breast, thyroid, astrocytoma, ACSL6, missense

## Abstract

Multiple non-related neoplasia does not have an established approach or benefits for performing whole-exome sequencing (WES) analysis. We report on a 46-year-old woman who developed astrocytoma, thyroid, and breast cancer within 10 years. The WES analysis found a novel missense variant in the *ACSL6* gene, and the protein modeling showed altered secondary and tertiary structures, which modify the binding to cofactors and substrates. ACSL6 is involved in lipid metabolism, expressed in the brain, thyroid, and breast tissues, and is associated with diverse types of cancer. Our study demonstrates the benefit of WES analysis compared with commercial panels in patients with non-related neoplasia.

## Introduction

Multiple related neoplasia occurs mostly in a hereditary cancer syndrome. Multiple primary malignancies (MPM) refers to a patient diagnosed with more than one primary malignancy and each tumor is unrelated histologically to the others (1). In this paper, we describe a patient with three multiple primary malignancies—breast, thyroid, and astrocytoma. MPM is extremely rare and only a few cases have been described in case reports. Although the incidence of MPM has increased in recent decades as the rate of survival in oncology patients has increased (2). In a retrospective study done from 2003 to 2009 in hospitalized patients, the Department of Oncology revealed that the incidence of MPM in 103 patients was 2.3% (2). In Ecuador, we lack studies on this concern, but the National Registry of Tumors has reported an increase in the incidence of breast and thyroid cancer among women from 1983 to 2013, which corresponds to two of the tumors diagnosed in our patient (3).

In clinical practice, physicians order commercial multiple neoplasia panels, which include the most common associated genes like BRCA1 DNA repair associated (BRCA1), ATM serine/threonine kinase (ATM), tumor protein p53 (TP53), and others. These genes tend to be associated with syndromic cancers or hereditary cancers but not with multiple primary tumors. In the case of non-related neoplasia or MPM, those panels are less useful and whole-exome sequencing (WES) is necessary.

We report on a 46-year-old woman who developed an astrocytoma, thyroid, and breast cancer within 10 years. We performed a WES analysis, modeled the protein, and concluded that commercial cancer panels could have missed the variant.

## Case Report

### Patient Information

**Figure 1:**
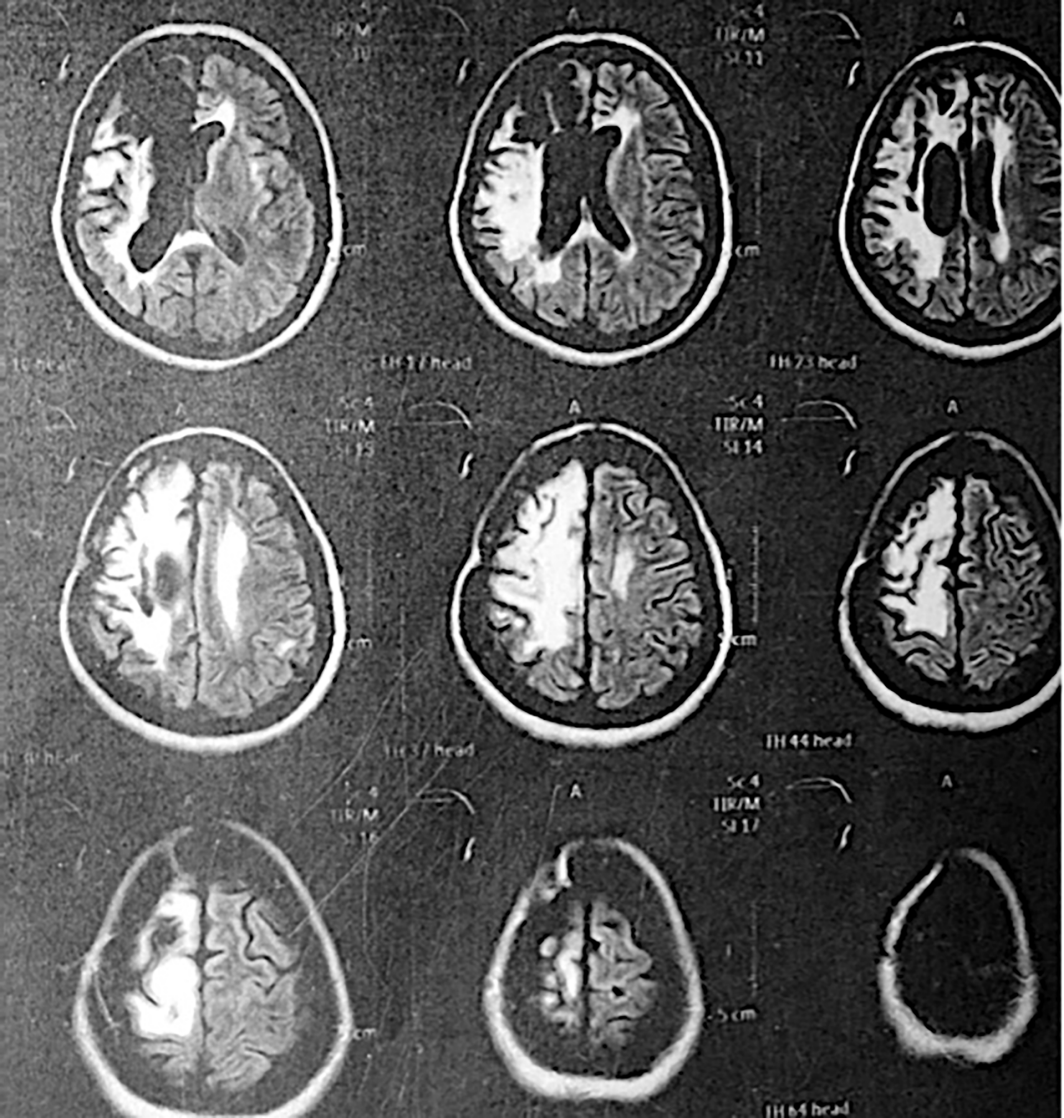
MRI of the patient years after surgery removal of the astrocytoma. An area of right frontoparietal encephalomalacia is observed with retraction of the frontal horn of the right lateral ventricle with slight perilesional edema.

We reported a 46-year-old Ecuadorian woman without a relevant family history of cancer or any other disease who came to our genetic outpatient clinic due to multiple non-related neoplasia. In 2010, she was diagnosed with a right frontotemporal GII astrocytoma. In 2013, she had a well-defined heterogeneous nodule (2.0 × 4.5 cm) in the left thyroid lobe. A biopsy of the mass confirmed classic papillary thyroid carcinoma in the left lobe. In 2017, during a routine exam, a mass was detected in the left upper quadrant of the left breast. Ultrasound showed a heterogeneous mass (52 × 38 mm) with pinpoint echogenic microcalcifications, lobed edges, peripheral vascularization, and ductal extension, classified as BIRADS 4C. The biopsy described a moderately differentiated, infiltrating ductal cancer, positive for the estrogen receptor 6, progesterone receptor 7, p53, and ki67. The patient was classified within the Karnofsky index with 60% functionality. In 2019, the patient came to our office with moderate dysarthria. A brain MRI showed gliosis in the left temporal region suggestive of metastasis without further investigation (**Figure 1**). Additional findings included a left spinal nodule with a lipomatous appearance and uterine fibroids (**Supplementary Figure 1**).

### Therapeutic Interventions

For managing astrocytoma, the patient underwent surgery, chemotherapy with temzolamide, and adjuvant radiotherapy for a year. Alternatively, to treat the classic papillary thyroid carcinoma in the left lobe, her thyroid was removed, and she received iodine-131 ablation. Since then, she has taken levothyroxine as a habitual medicine. Lastly, for managing ductal cancer, the patient underwent a modified left radical mastectomy with removal of the axillary nodes because of micro-metastases. Subsequently, the patient was treated with four cycles of chemotherapy with the AC scheme (doxorubicin and cyclophosphamide), paclitaxel, and adjuvant radiotherapy for 4 months.

After the patient was treated for astrocytoma, she developed left hemiparesis and seizures, which have been treated to date with carbamazepine. In 2015, brain magnetic resonance imaging (MRI) was performed due to a complaint of headache and visual distortion. An area of encephalomalacia in the right frontoparietal lobe with retraction of the frontal horn of the lateral ventricle and mild perilesional edema were found, probably secondary to the radiotherapy treatment in 2010. Besides the mentioned clinical findings, no other side effects have been found to date in the patient.

### Molecular Analysis

We performed a WES analysis on the peripheral blood of the patient To analyze the origins of the non-related neoplasia. We found 697 SNVs exonic non-synonymous variants and omitted any synonymous variation. We identified a transition from guanine to adenine (c.G451A) in the fifth exon of the acyl-CoA synthetase long chain family member 6 (ACSL6) gene, resulting in a change from valine to methionine (p.V151M). The variant is a nonsynonymous variation with a 0.0001 frequency in the Genome Aggregation Database (gnomAD), 0.002 in 1,000 g, and 0.0007 in The Exome Aggregation Consortium (ExAC) databases. Protein damage algorithms showed SIFT score = 0.001 (damaging), Polyphen score = 0.899 (deleterious), LRT score = 0, mutation taster = 1, FATHMM_score = 1.47, and PROVEAN_score = −2.39. Commercial panels for multiple neoplasia include genes such as BRCA1/2, ATM, TP53, and others (**Supplementary Tables 1**, **2**). None of these genes were altered in the sample.

### Protein Modeling

**Figure 2:**
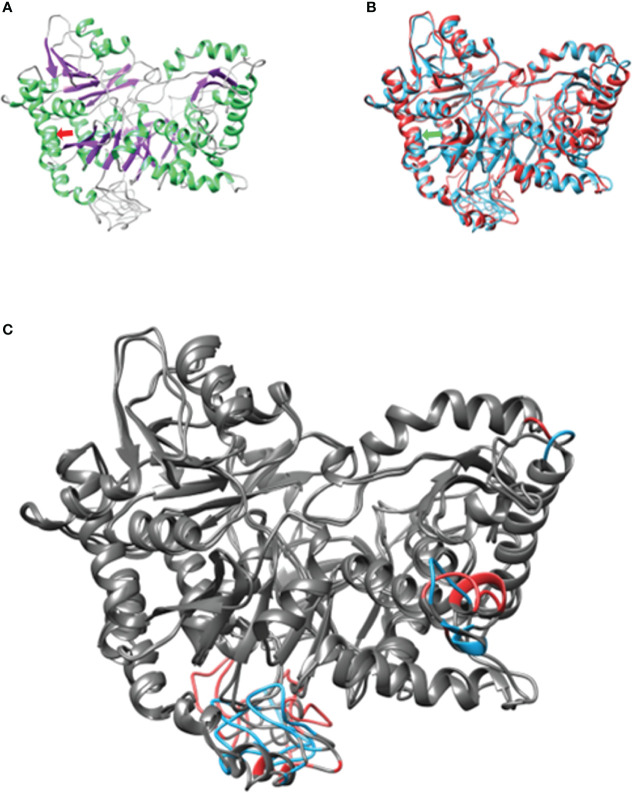
Structural conformation and pairing of ACSL6. **(A)** Weight isoform with secondary structure coloring. On the light green α-helices, on the purple β-sheet and the coils. **(B)** Structural alignment of both models. In both arrows they represent the point where the amino acid change occurs. **(C)** Differences representing 21.5%. dissimilarity. At the bottom, the transmembrane domain. In gray structural agreements between models.

To confirm the protein damage, protein modeling was performed using the Needleman–Wunch algorithm and the BLOSUM 62 matrix to compare the secondary structures of the normal and mutant proteins (patient). Both proteins had an 88.52 similarity. However, the mutant differed within and between the secondary structure angles and had a modification in the infrastructural bonds of the anterior (<125) and posterior (<125) amino acids (>127) (4–6). Modifications at this level alter how enzymes—like ACSL6—carry out their catalysis, how atoms bind to cofactors, and how substrates interact with the binding pocket (**Figure 2**) (4, 7) (**Supplementary Figure 2**). In the tertiary structure, the variation damages the enzyme energy and mechanical performance (8). At a biochemical level, switching valine with methionine is supposed to be a minimal alteration, as these two amino acids are characterized as non-polar aliphatic. In practice, we can see how this minimal change alters the tertiary structure, resulting in differences in the molecular performance at an energetic and mechanical level. Valine poses an isopropyl group as a lateral chain, a poor-binding prosthetic group. On the other hand, methyl presents an S-methyl thio-ester group, the same that could act as an antioxidant when it is inside the structure of a protein. It is well known that methionine presenting a sulfuric group could carry out weak interactions besides the polar state that the same ion could display. As can be seen in the one-on-one neighborhood, a binding amino acid neighborhood of the 126-residue position in both cases presents a different configuration of their intra-structural bonds (9). ACSL6 works in environments with high concentrations of ions and molecules such as free radicals due to its catalytic metabolism rich in oxidation–reduction reactions for synthesis and decomposition. It is in this way that proteins that localize in such environments need the specific organization of their structure, like a tighter core and a certain degree of flexibility in their junctions. As we observe, the mutant protein is characterized by a 21.5% dissimilarity when compared with the WT, with differences mainly observed between junctions. These findings allow us to interpret that the V126M mutant isoform presents a structural change with a high degree of relevance, being an important discovery for the correct characterization of many genetic diseases and other illnesses subcategorized as variants of uncertain significance like this (9, 10).

## Discussion

Common cancer analysis includes the use of commercial cancer panels that include traditional genes like BRCA. However, there is a lack of an established approach for non-related neoplasia, and in these cases, traditional panels exclude other non-traditional cancer genes (11). In relation to that debate, we demonstrate that in non-related neoplasia, other non-traditional genes should be considered based on our case report in which we performed a whole-exome sequencing analysis, identified a new variant in a non-traditional cancer gene, modeled the mutant protein, and concluded that commercial cancer panels could have missed the variant.

The *ACSL6* gene is located on chromosome 5q31.1 and is expressed mainly in the frontal cortex, the anterior cingulate cortex (mainly in astrocytes), and to a lesser extent in tissues such as thyroid, breast, erythrocytes, and leukocytes, among others. This gene is responsible for catalyzing the conversion of long-chain fatty acids to their active form, acyl-CoA, for lipid synthesis and degradation through beta oxidation. In the brain, this gene maintains adequate levels of docosahexaenoic acid (DHA) that are important for neuronal membrane fluidity, neuronal survival, and regulation of neuroinflammation (12).

The long chain fatty acid synthase (ACSL) gene family has been linked to the evolution and prognosis of different types of cancer. While *ACSL1* is associated with colorectal, brain, esophageal, liver, breast, leukemia, and sarcoma cancers, *ACSL3* is overexpressed in squamous lung cancer and downregulated in prostate and colorectal cancer. The expression of *ACSL4* correlates with hormonal sensitivity in breast and prostate cancer and is over-expressed in liver cancer. *ACSL5* is used as a marker of villi atrophy in the gastrointestinal tract, and in colorectal cancer it regulates the lipid metabolism dependent on TP53. The overexpression of *ACSL5* correlates with a better prognosis in breast cancer, but the overexpression in the brain results in a worse prognosis (13, 14). ACSL6 is under-expressed in leukemia, brain tumors, and cervical cancer (13). According to the cBioPortal for Cancer Genomics database, ACSL6 is mutated in 0.2% of thyroid cancers, 0.52% of astrocytomas, and 0.51% of invasive ductal carcinomas of the breast.

ACSL promotes cell growth, facilitates tumor invasion, and prevents apoptosis. The interaction between fatty acids and ACSL results in an anabolic pathway of lipid biosynthesis and a catabolic pathway of beta oxidation of fatty acids depending on the tissue composition (14). Fatty acids derived from the diet activate different transcription factors, which bind to the promoters of different isoforms of ACSL. Cancer cells epigenetically upregulate ACSL, increase ACSL transcription by oncogenes, and stabilize ACSL mRNA. Fatty acids and high levels of ACLS provide functional and biosynthetic intermediates that promote cell proliferation without checkpoints. The interaction between overexpression of ACSL by cancer cells and ACSL preventing evolution becomes a vicious cycle of neoplasia and metastasis.

We suggest a WES analysis approach in patients with multiple non-related neoplasia like ours, compared to commercial genetic cancer panels, which all exclude ACSL6. The commercial panels have associated oncological genes that function as a diagnostic method for different hereditary syndromes and use the previously described data for common or related neoplasia like Multiple Endocrine Neoplasia (MEN) or Li-Fraumeni syndrome. Additionally, WES analysis provides supplementary non-traditional genes, is cost-effective for patients with multiple non-related neoplasia, and could open the door to personalized medicine and the development of new diagnosis and therapeutic approaches targeting the affected gene, since currently there are no targeted therapies against this gene

In one study, 3,040 WES analyses were conducted for different clinical cases, and it showed that the use of WES facilitates the identification of novel candidate genes since 24.2% of the genes identified were later reported as pathogenic variants related to the definitive diagnosis (15).

Once again, proving the clinical benefits of WES analysis in the detection of novel pathological variants, it was documented that in a study of 97 patients who had tumors, 91 cases (94%) gave informative results, and it was also seen that the WES contributed to the identification of the pharmacological alterations of the approved drugs and therapies in clinical or preclinical (16). In conclusion, it is evident that WES analysis is a method that helps recognize pathogenic variants that may impact oncological diseases and additionally helps identify predictive biomarker candidates for response to certain drugs.

### Conclusion

In conclusion, we report a patient with astrocytoma, thyroid, and breast cancer caused by a missense variation in the ACSL6 gene, which alters the secondary and tertiary structure and dysregulates lipid metabolism. Common cancer panels missed the variant; therefore, we suggest WES for non-related neoplasia instead of commercial panels.

## Data Availability Statement

The original contributions presented in the study are included in the article/**Supplementary Material**. Further inquiries can be directed to the corresponding author.

## Ethics Statement

The studies involving human participants were reviewed and approved by the Universidad San Francisco de Quito Comité de Ética. The patients/participants provided their written informed consent to participate in this study.

## Author Contributions

MC and EF analyzed the WES results, performed a literature review of the neoplasia, and wrote the paper. BA performed the protein modeling and wrote the paper. VR contacted the patient, provided genetic counseling, performed the literature review, analyzed the WES results, and wrote the paper. KH performed the WES analysis. CR contacted the patient and provided genetic counseling. All authors listed have made a substantial, direct, and intellectual contribution to the work and approved it for publication.

## Conflict of Interest

The authors declare that the research was conducted in the absence of any commercial or financial relationships that could be construed as a potential conflict of interest.

## Publisher’s Note

All claims expressed in this article are solely those of the authors and do not necessarily represent those of their affiliated organizations, or those of the publisher, the editors and the reviewers. Any product that may be evaluated in this article, or claim that may be made by its manufacturer, is not guaranteed or endorsed by the publisher.
