# Supplementary material for: Novel Variation in Acyl-CoA Synthetase Long Chain Family Member 6 (ACSL6) Results in Protein Structural Modification and Multiple Non-Related Neoplasia in a 46-Year-Old: Case Report
Source: Front Oncol. 2022 Jun 2;12:899579. doi: 10.3389/fonc.2022.899579 (PMC9215171; doi:10.3389/fonc.2022.899579)
Supplement: Supplementary file 1 [file DataSheet_1.docx]

**Supplementary data**

**Supplementary figure 1.- TimeLine.** shows the consecutive important events in this case which demonstrate the improbable case of metastasis by each cancer since each was successfully treated before the other one developed.


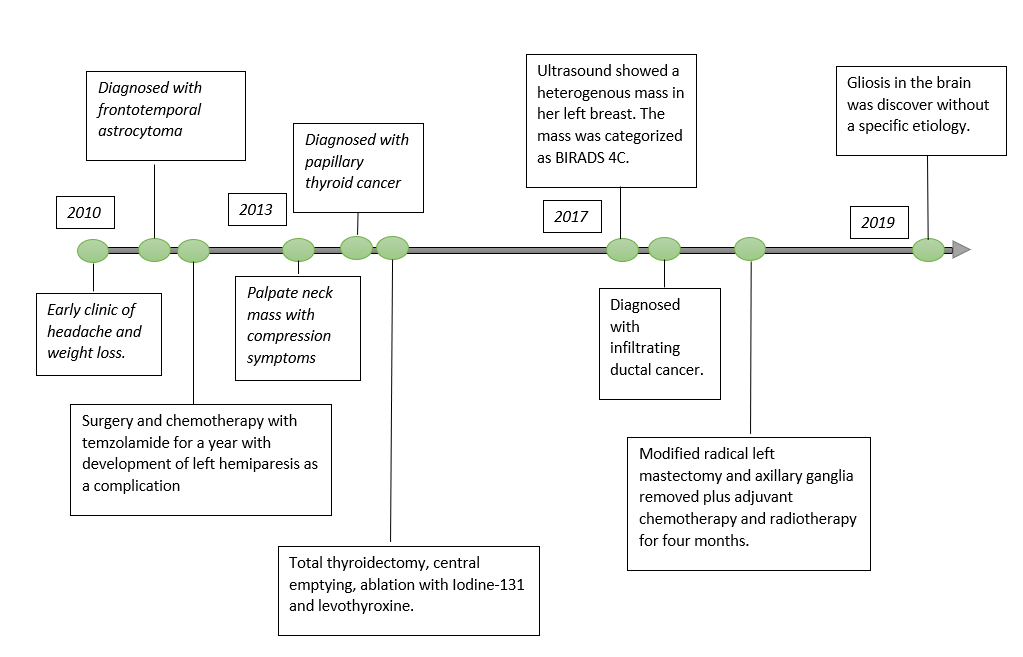


**Supplementary figure 2.- Sequence alignment of Wt and mutant V126M**

Description: Note the identity of both sequences except for the 126 positions. In addition, both amino acids have the same biochemical properties as apolar aliphatic amino acids.


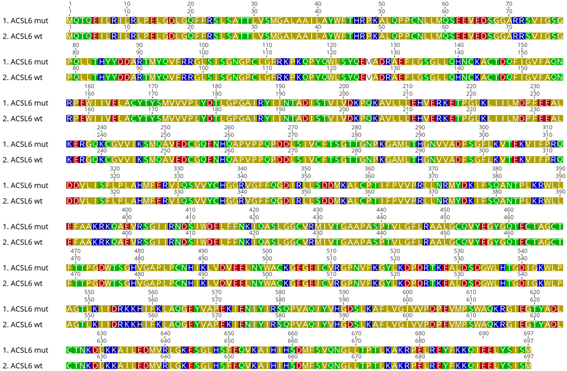
**Supplementary figure 2**.- a) and b) Wt of structural neighborhood and mutant M2171V respectively.

Description: It is observed how the intrahelical and structural hydrogen bonds change when compared. The side chain represented at the atomic level is the position where the mutation occurs.


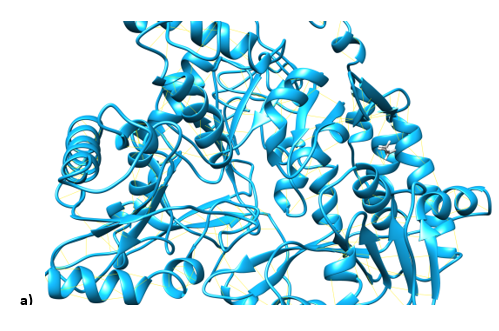

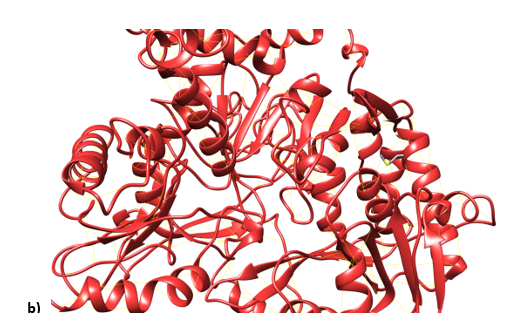


**Supplementary Table 1.- Genes associated with low grade astrocytoma, thyroid gland papillary carcinoma and breast ductal carcinoma.**

Genes associated with these cancers were retrieved from the Malacards database. All the genes were searched in the patient WES analysis and none of them were found to be related to the condition.

| ***Tumor*** | ***Associated genes*** |
| --- | --- |
| **Low Grade Astrocytoma** | TP53, BCAN, CDKN1B, CDKN2A, CDKN2B, HSPB1, IDH1, IL13, IL13RA1, IL12RA2, NCAN, NOVA1, PTGDS, SCN1A, SCN2A, SCN3A, SCN8A, SOX2, STAT6, TNC. |
| **Thyroid Gland Papillary Carcinoma** | BRAFF, CRNDE, ELF-AS1, GAS5, GAS8-AS1, HAGLR, LINC00271, LINC01929, LINC02471, LNCRNA-ATB |
| **Breast Ductal Carcinoma** | RAD54L, ZFAS1, ERBB2, ESR1, TP53, CDH1, PGR, PIP, EGFR, CCND1, CTSD, VEGFD, TFF1, BRCA1, KRT8, MKI67, CTNNB1, CDH3, NME1, FAP, AR, KRT19, MET, CD44, VEGFC, CDKN1A, BRCA2, MMP26, SMAD4, THBS1, MUC1, KRT14, TIMP2, KRT5, ESR2, MIR17, PLAU, CYP19A1, BCL2, KRT7, TP64, SERPINB5, MUC5AC, CD24, MMP9, VEGFA, S100A7, SYP |

**Supplementary table 2.- Common commercial hereditary cancer panels**

| Commercial hereditary cancer panel | Genes tested |
| --- | --- |
| Panel N.1 (invitae) | AIP. ALK, APC, ATM, AXIN2, BAP1, BARD, BLM, BMPR1A, BRCA1, BRCA2, BRIP1, CASR, CDC73, CDH1, CDK4, CDKN1B CDKN1C, CDKN2A, CEBPA, CHEK2, CTNNA1, DICER1, DIS3L2, EGFR, EPCAM, FH,FLCN, GATA2, GPC3, GREM1, HOXB13, HRAS, KIT, MAX, MEN1, MET, MITF, MLH1, MSH2, MSH3, MSH6, MUTYH, NBN, NF1, NF2, NTHL1, PALB2, PDGFRA, PHOX2B, PMS2, POLD1, POLE, POT1, PRKAR1A, PTCH1, PTEN, RAD50, RAD51C, RAD51D, RB1, RECQL4, RET, RUNX1, SDHA, SDHAF2, SDHB, SDHC, SDHD, SMAD4, SMARCA4, SMARCB1, SMARCE1, STK11, SUFU, TERC, TERT, TMEM127, TP53, TSC1, TSC2, VHL, WRN, WT1. |
| Panel N.2 (Custom Next Cancer) | AIP, ALK, APC, ATM, AXIN2, BAP1, BARD1, BLM, BMPR1A, BRCA1, BRCA2, BRIP1, CASR, CDC73, CDH1, CDK4, CDKN1B, CDKN2A, CFTR, CHEK2, CPA1, CTNNA1, CTRC, DICER1, EGFR, EGLN1, EPCAM, FAM175A, FANCC, FH, FLCN, GALNT12, GREM1, HOXB13, KIF1B, KIT, LZTR1, MAX, MEN1, MET, MITF, MLH1, MLH3, MRE11A, MSH2, MSH3, MSH6, MUTYH, NBN, NF1, NF2, NTHL1, PALB2, PALLD, PDGFRA, PHOX2B, PMS2, POLD1, POLE, POT1, PRKAR1A, PRSS1, PTCH1, PTEN, RAD50, RAD51C, RAD51D, RB1, RECQL, RET, RINT1, RPS20, SDHA, SDHAF2, SDHB, SDHC, SDHD, SMAD4, SMARCA4, SMARCB1, SMARCE1, SPINK1, STK11, SUFU, TERT, TMEM127, TP53, TSC1, TSC2, VHL & XRCC2 |
| Panel N.3 (Cancer Next Expanded) | *AIP, ALK, APC, ATM, AXIN2, BAP1, BARD1, BLM, BMPR1A, BRCA1, BRCA2, BRIP1*  *CDC73, CDH1, CDK4, CDKN1B, CDKN2A, CHEK2, CTNNA1, DICER1, EGFR, EGLN1*  *EPCAM, FANCC, FH, FLCN, GALNT12, GREM1, HOXB13, KIF1B, KIT, LZTR1, MAX, MEN1, MET, MITF, MLH1, MSH2, MSH3, MSH6, MUTYH, NBN, NF1, NF2, NTHL1, PALB2, PDGFRA, PHOX2B, PMS2, POLD1, POLE, POT1, PRKAR1A, PTCH1, PTEN*  *RAD51C, RAD51D, RB1, RECQL, RET*  *SDHA, SDHAF2, SDHB, SDHC, SDHD, SMAD4, SMARCA4, SMARCB1, SMARCE1, STK11, SUFU, TMEM127, TP53, TSC1, TSC2, VHL & XRCC2* |
| Panel N.4 (Cancer Next) | *APC, ATM, AXIN2, BARD1, BMPR1A, BRCA1, BRCA2, BRIP1, CDH1, CDK4, CDKN2A, CHEK2, DICER1, EPCAM, GREM1, HOXB13, MLH1, MSH2, MSH3, MSH6, MUTYH, NBN, NF1, NTHL1, PALB2, PMS2, POLD1, POLE, PTEN, RAD51C, RAD51D, RECQL, SMAD4, SMARCA4, STK11 & TP53* |
| Panel N.5 (CentoCancer) | *ABRAXAS1, APC, ATM, AXIN2, BAP1, BARD1, BLM, BMPR1A, BRCA1, BRCA2, BRIP1, CDH1, CDK4, CDKN2A, CHEK2, DICER1, DIS3L2, EPCAM, FANCC, FH, FLCN, GALNT12, HNF1B, HOXB13, KIT, MC1R, MEN1, MET, MITF, MLH1, MLH3, MRE11, MSH2, MSH3, MSH6, MUTYH, NBN, NF1, NTHL1, PALB2, PMS1, PMS2, POLD1, POLE, POT1, PRSS1, PTCH1, PTEN, RAD50, RAD51C, RAD51D, RECQL, RET, RNF43, SDHA, SDHAF2, SDHB, SDHC, SDHD, SMAD4, SMARCA4, STK11, TGFBR2, TP53, TSC1, TSC2, VHL, WT1, XRCC2 & XRCC3* |
| Panel N.6 (NATERA) | BRCA1, BRCA2, ATM, BRIP1, CDH1, CHEK2, EPCAM, MLH1, MSH2, MSH6, NBN, NF1, PALB2, PMS2, PTEN, RAD51C, RAD51D, STK11, TP53, APC, AXIN2, BAP1, BARD1, BMPR1A, CDK4, CDKN2A, GALNT12, GREN1, HOXB13, MEN1, MITF, MSH3, MUTYH, NTHL1, POLD1, POLE, RNF43, RPS20, SMAD4, VHL, CTNNA1, DICER1, KIT, MRE11A, PDGFRA, RAD50, SDHA, SDHB, SDHC, SDHD, SMARCA4, TSC1 & TSC2. |
| Panel N.7 (United Healthcare) | BRCA1, BRCA2, CHEK2, PALB2, BRIP1, RAD51C, PTEN, TP53, STK11, NBN, ATM, CDH1, APC, EPCAM, MLH1, MSH2, MSH6, PMS1, PMS2, STK11, CDH1, BMPRIA, SMAD4, POLD1, GREM1 & POLE. |
| Panel N.8 (Life Labs genetics) | APC, ATM, AXIN2, BARD1, BMPR1A, BRCA1, BRCA2, BRIP1, CDH1, CDKN2A (p14ARF), CDKN2A (p16INK4a), CHEK2, DICER1, EPCAM, GREM1, KIT, MEN1, MLH1, MSH2, MSH6, MUTYH, NBN, NF1, PALB2, PDGFRA, PMS2, POLD1, POLE, PTEN, RAD50, RAD51C, RAD51D, SDHB, SDHC, SDHD, SMAD4, SMARCA4, STK11, TP53, TSC1, TSC2 & VHL. |
| Panel N.9 (Gen Dx) | APC, ATM, AXIN2, BAP1, BARD1, BMPR1A, BRCA1, BRCA2, BRIP1, CDH1, CDK4, CDKN2A, CHEK2, EPCAM, FANCC, FANCM, FH, FLCN, HOXB13, MET, MITF, MLH1, MSH2, MSH6, MUTYH, NBN, NF1, NTHL1, PALB2, PMS2, POLD1, POLE, POT1, PTEN, RAD51C, RAD51D, RECQL, SCG5/GREM1, SDHB, SDHC, SDHD, SMAD4, STK11, TP53, TSC1, TSC2 & VHL |
| Panel N.10 (GERMLINE) | BRCA1, BRCA2, p53, PTEN, COH1, MLH1, MSH2, MSH6, PMS2, MYH, APC, STK11, MSI, RNASEL, ELAC2, MSR1, PALB2, PRSS1, SPINK1 & COKN2A. |
